# A *bona fide* MAP kinase

**DOI:** 10.1016/j.jbc.2022.102432

**Published:** 2022-08-28

**Authors:** Laurel Oldach

Yeast and humans have a few rather noticeable differences. Yet many details of human biochemistry were first worked out in yeast, insights inherited from the days when yeast cells were much more genetically tractable than human cells. Then, the challenge for human biologists was to determine which findings could be translated from yeast and which were not shared between fungi and mammals.

The mitogen-activated protein kinases, or MAPKs, are an example. Initially identified in yeast, these enzymes respond to growth factors and cytokines recognized by receptor tyrosine kinases, along with physiological stressors, and regulate processes in growth, proliferation, and response to stress. They act in multiple-kinase cascades: a MAP kinase kinase kinase activates a MAP kinase kinase, which in turn activates the MAPK itself, which phosphorylates transcription factors and other substrates. The avalanche of enzymatic activation amplifies what may be a small initial stimulus into a robust cellular response (1).

The enzymes at the effector end of the cascade, MAPKs, all share an activation loop that can be phosphorylated on closely juxtaposed tyrosine and threonine residues. Various MAPKs are activated under different circumstances and activate different transcription factors—yet sometimes, researchers observed in the 1990s, their activity would overlap, creating responses in yeast that seemed to depend on numerous MAPKs (2). It was unclear which of those complex findings from yeast—if any—would translate to mammalian biology.

After the first MAPK was found in human cells, University of Massachusetts Medical School professor Roger Davis said, researchers reasoned that if yeast had numerous MAPKs, humans might have many as well (3). Davis said, “There was essentially an explosion of cloning.”

The kinase p38 was identified as part of that explosion (4, 5). p38 showed high homology to a yeast kinase called HOG1 and shared some features with known MAPKs, but it also had a slightly unusual activation loop sequence. Davis said, “The big question was, is it truly a MAPK?”

Davis, whose lab studied the related Jun kinase, or JNK, at the time, quickly became interested in p38 because the two enzymes responded to similar stimuli, such as inflammatory cytokines, heat and osmotic shock, and UV irradiation. A classic article that Davis’s lab published in the *Journal of Biological Chemistry* in 1995 helped to establish that p38 was genuinely a MAPK (6).

Through careful biochemical characterization, the lab demonstrated that p38 was phosphorylated on both tyrosine and threonine residues in a three amino acid activation loop—a key feature of MAPK biology (7). They also identified a substrate, the transcription factor ATF2, allowing them to develop a functional assay to study p38 activity. With that ability in hand, they examined the differences between p38 and JNK activity in response to stressors such as UV irradiation and endotoxin.

First author Joel Raingeaud said, “We were expecting in terms of specificity of target—the transcription factor—or time course of activation, it would be a bit different.”

Indeed, they found that the two kinases had distinct activation kinetics, suggesting that p38 had a place of its own in the tangled wiring diagram of MAPK signaling. Around the same time, the lab also demonstrated that the activation pathways for p38, JNK, and Erk are separate and generally independent, using different kinase kinases (8). Davis said, “They are clearly separate pathways, but they also have overlapping substrates.”

Having established some commonalities p38 shared with its fellow MAPKs and some differences that distinguished them, the lab published its findings just 11 months after the discovery of p38 had been reported. However, Davis said the study was not completed in any particular rush “At the time, we were good at doing exactly what was described in that paper,” he said. “Every method and technique was very routine in the laboratory.”

In the ensuing decades, researchers have come to appreciate even greater complexity in the MAPK pathways. For example, human cells have 14 MAPKs, including four genes encoding variants of p38 and three JNKs (9). Additional isoforms of each kinase are produced through alternative splicing, while tissue-specific expression patterns, docking sites, and scaffolding proteins regulate interactions between kinases and their substrates (10).

“The biochemistry gets quite complicated—but it also provides opportunities,” Davis said. That is, if specific isoforms or protein–protein interactions are only important in specific cell types or signaling contexts, they may be targets for treating disease. Davis’s lab now focuses on JNK and its contribution to inflammation related to metabolic disease and cancer. The lab is interested in understanding what differentiates the two kinase populations and finding treatment strategies that exploit those differences.

Davis received a phone call from a citation tracking service a few years after the article was published. The article had been so widely cited, they said, that they wondered why he had published in the *Journal of Biological Chemistry*. He said he told them, “This was a perfect JBC paper… and it was published in exactly the right journal for the right audience.”
